# Analysis of Chlorine

**Published:** 1879-11

**Authors:** 


### ANALYSIS OF CHLORINE.

Scheele, the discoverer of chlorine, supposed it was a compound body, and only when all attempts failed to decompose it, was it placed among the elementary bodies. Recently Prof. Victor Meyer, of Zurich, has succeeded in separating it into other constituents. He uses an extremely high temperature, 157° Cent., and the manipulations necessary to testing under these conditions are as yet so imperfect that he has not been able to say whether the resultant is oxygen or some other element. He submitted to the same temperature oxygen, sulphur and quicksilver, but they underwent no alteration. Iodine behaved like chlorine, showing itself to be a compound body.

